# Overexpression of Wip1 Is Associated with Biologic Behavior in Human Clear Cell Renal Cell Carcinoma

**DOI:** 10.1371/journal.pone.0110218

**Published:** 2014-10-15

**Authors:** Sulai Liu, Lin Qi, Weqing Han, Xinxing Wan, Shusuan Jiang, Yuan Li, Yu Xie, Longfei Liu, Fuhua Zeng, Zhizhong Liu, Xiongbing Zu

**Affiliations:** 1 Department of Urology, Xiangya Hospital, The Central South University, Changsha, Hunan, China; 2 Department of Urology, The Affiliated Tumor Hospital of Xiangya Medical School, The Central South University, Changsha, China; 3 Department of Endocrinology, Third Xiangya Hospital, Central South University, Changsha, China; Baylor College of Medicine, United States of America

## Abstract

Wild-type p53-induced phosphatase (Wip1 or PPM1D) has been reported to be aberrantly expressed in various cancers and correlated with the malignant behavior of cancer cells. However, the function of Wip1 in RCC remains unclear. The present study investigated its abnormal expression and dysfunctions in clear cell renal cell carcinoma (ccRCC) in vitro. With the combination of immunohistochemistry, western blotting, immunofluorescence, qRT-PCR, and cell proliferation, migration and invasion assays, we found that levels of Wip1 mRNA and protein were dramatically increased in human ccRCC tissues (P<0.001 for both), and upregulation of Wip1 was significantly associated with depth of invasion (P<0.001), Distant metastasis (P = 0.001), lymph node status (P<0.001) and Fuhrman grade (P<0.001). Wip1 knockdown inhibited the proliferation, migration and invasion of 786-O and RLC-310 cells, whereas Wip1 overexpression promoted the growth and aggressive phenotype of 786-O and RLC-310 cells in vitro. The uni- and multivariate analyses indicated that expression of Wip1 was an independent predictor for survival of ccRCC patients (P = 0.003, P = 0.027 respectively). Wip1- negative patients had a higher tumor-free/overall survival rate than patients with high Wip1 expression (P = 0.001, P = 0.002 respectively). Overexpression of Wip1 is useful in the prediction of survival in ccRCC patients.

## Introduction

Renal cell carcinoma (RCC) is the most prevalent malignancy of the adult kidney, accounting for approximately 90–95% of all kidney neoplasms [1], with annual estimations of 209,000 new cases and 102,000 cancer-related deaths in the world [2]. Clear cell renal cell carcinoma (ccRCC) represents the predominant subtype of RCC and constitutes for approximately 75–80% of all cases [3]. Surgery is the most effective treatment of RCC, while chemotherapy and radiotherapy are not as successful in the control of RCC. However, approximately a quarter of RCC clinic patients will develop a metastatic disease despite curative surgical removal of the primary tumor [4]. The overall five-year survival rate of patient with RCC ranges from 5% to 10%, with a median survival of only about 13 months [5]. Therefore, it is of paramount importance to better understand the molecular mechanisms involved in the initiation and progression of RCC. Identification of novel biomarkers associated with disease progression and metastasis of RCC and combination of their application with traditional diagnostic and prognostic parameters would contribute to development of effective strategies for the prevention, early diagnosis and treatment of RCC.

Wild-type p53-induced protein phosphatase 1 [(Wip1 or protein phosphatase, Mg^2^^+^/Mn^2+^ dependent, 1D (PPM1D)] is a member of the protein phosphatase type 2C (PP2C) and p53 target gene family [6], [7]. Wip1 is activated by p38 mitogen-activated protein kinase (MAPK) and the p53 pathway in response to various stresses, including UV, γ-radiation, and alkylating agents. Activated Wip1 directly dephosphory-lates checkpoint kinase 1 (Chk1), checkpoint kinase 2 (Chk2), p38 MAPK, uracil DNA glycosylase (UNG), ataxia-telangiectasia-mutated (ATM) kinase, mdm2, g-H2AX, and p53 [8], [9], suggesting its role as a homeostatic regulator that reverses protein kinase cascades such that damaged cells are induced to reenter the normal cell cycle after completing DNA repair. Moreover, the Wip1 gene is frequently amplified/overexpressed in human cancers, including breast cancers, ovarian clear-cell adenocarcinomas, pancreatic neuroendocrine tumors, neuroblastomas, and pancreatic cancers, all of which rarely carry a p53 mutation [10], [11], [12]. Recently, Sun GG et al. [13] have reported that Wip1 protein was increased in kidney carcinoma. However, Wip1 expression and its clinical significance in RCC remain unclear. In this study we evaluated Wip1 expression in clinical ccRCC tissue specimens, determined its correlation with clinicopathological characteristics and assessed the influence of expression of Wip1 on the survival of patients with RCC. Furthermore, we investigate the roles of Wip1 expression in biologic behavior of renal cancer cells.

## Material and Methods

### Patients and Tissue Source

One hundred and sixteen primary renal cancer and distant normal tissue samples were obtained from ccRCC patients that underwent radical or partial nephrectomy between August 2005 and September 2007 at The Department of Urology, Xiangya Hospital and The Affiliated Tumor Hospital of Xiangya Medical School, The Central South University. None of the patients received any preoperative chemo- and radiotherapy, or other medical interventions. The patients consisted of 66 men and 50 women (mean age, 54.2 years; ranging between 27 and 81 years). All cases were pathologically diagnosed to have ccRCC and staged in accordance with the latest Tumor-Node-Metastasis (TNM) classification system. The clinicopathological characteristics of the patients were retrieved from the medical records and are summarized in table 1. The fresh specimens of tumor tissue or adjacent normal epithelium 1 cm apart from the tumor edge were immediately taken after the surgery, one was fixed in 4% paraformaldehyde solution, and then embedded in paraffin for immunohistochemistry, and the other one was stored in liquid nitrogen for RT-PCR and Western blot assay. Follow-up data were obtained by phone interview, postal letter communication, and the outpatient clinical database. All patients were monitored from the date of initial surgery until death, or the closing date of this study (November 30, 2011). The mean follow-up period of time was 50.2 months (range between 6 and 62 months). This study was reviewed and approved by the Ethics Committee of The Central South University, and an informed consent form was signed by each patient before surgery.

**Table 1:** Clinicohistopathologic characteristics of patients with ccRCC and their associations with Wip1 expression.

Characteristics	All cases	Wip1 expression (n,%)		P-value
		High(n = 77)	Low(n = 39)	
Age(yers)				0.194
<50	72	51(70.8)	21(29.1)	
≥50	44	26(59.1)	18(40.9)	
gender				0.239
Male	66	49(74.2)	21(31.8)	
Female	50	32(64)	18(36)	
Tumor size (cm)				0.948
≤4.0	44	30(68.1)	14(31.8)	
4.1∼7.0	32	21(65.6)	11(34.4)	
>7.1	40	26(65)	14(35)	
Depth of invasion				**<0.001**
T1+T2	77	60(77.9)	17(22.1)	
T3+T4	39	17(43.6)	22(56.4)	
Symptoms of diagnosis				0.327
Incidental	52	37(71.2)	15(28.8)	
Symptoms	64	40(62.5)	24(37.5)	
Fuhrman grade				**<0.001**
G1–2	88	71(80.7)	17(19.3)	
G3–4	28	6(21.4)	22(78.6)	
TNM stage				0.806
I+II	82	55(67.1)	27(32.9)	
III+IV	34	22(64.7)	12(35.3)	
Lymph node status				**<0.001**
Negative	94	73(77.7)	21(22.3)	
Positive	22	4(18.2)	18(81.8)	
Distant metastasis				**0.001**
Absent	98	71(72.4)	27(27.6)	
Present	18	6(33.3)	12(66.7)	

### Evaluation of Immunohistochemical Staining

The immunohistochemical staining of Wip1 on tumor cells was evaluated in accordance with our previous reports [14], [15]. In brief, the immunohistochemical staining was randomly scored by two independent investigators in a blinded fashion, based on the intensity and percentage of cells with Wip1 staining. Referring to the predominant intensity, staining intensity was denoted as 0 (negative), 1 (weak), 2 (moderate) or 3 (strong). The score of staining density was given according to the percentage of positive staining cells as follows: 0, less than 5%; 1, 5 to 25%; 2, 25 to 50%; or 3, more than 50%. The final score was then calculated by adding the two above scores, and scores of 0–2 were considered as low expressions while scores of 3–6 were defined as high expressions.

### Cell Culture and Transfection

Human RCC cell line 786-O, RLC-310 and immortalized normal human proximal tubule epithelial cell line HK-2 were cultured as described by Cheng et al. [16]. All cell lines were preserved in our laboratory (Central Laboratory of Xiangya Hospital, Xiangya Medical College, CSU, Hunan, China). In accordance with Wip1 gene sequence in the NCBI database and siRNA design principles, the Wip1 expression vector pcDNA3.1-Wip1, pcDNA3.1-control and Wip1-specific shRNAs were designed and synthesized by Shanghai ShengGong Co., Ltd., whose sequences were as follows: sense strand 5′-CCAAUGAAGAUGAGUUAUAdTdT- 3′, a ntisense strand 3 ′ -dTdTGGUUACUUCUACUCAAUAU-5 ′, and target sequence CCAATGAAGATGAGTTATA. In addition, a negative shRNA control that shared no homology to siRNA–Wip1 genome sequence was designed and synthesized. And then transfected into 60%-confluent 786-O, RLC-310 and HK-2 cells, respectively, using lipofectamin 2000 (Invitrogen, Carlsbad, CA, USA) according to the manufacturer's instructions.

### Migration Assay

Cell migration was determined by using a modified two chamber migration assay with a pore size of 8 mm. For migration assay, 2*10^5^ 786-O and RLC-310 cells were seeded in serum-free medium in the upper chamber. After 12 h incubation at 37°C, cells in the upper chamber were carefully removed with a cotton swab and the cells that had traversed the membrane were fixed in methanol and stained with leucocrystal violet. The number of invasive cells was determined by counting the leucocrystal violet-stained cells. For quantification, cells were counted under a microscope in five fields (up, down, median, left, right.*200).

### Invasion Assay

The invasion assay was performed using a modified two chamber plates with a pore size of 8 mm. The transwell filter inserts were coated with matrigel (BD Biosciences, NJ, USA).1*10^5^ 786-O and 1*10^5^ RLC-310 cells were seeded in serum-free medium in the upper chamber. After 24 h incubation at 37°C, noinvasive cells were gently removed from the top of the matrigel with a cotton-tipped swab. Invasive cells at the bottom of the matrigel were fixed in methanol, stained with leucocrystal violet and counted.

### Cell Proliferation Assay

The cell growth rates were detected using a CCK-8 cell proliferation assay (CCK-8 kit; Boster Ltd., Wuhan, China) according to the manufacturer's instructions. In brief, the cells (4*10^3^/well) were seeded in a 96-well plate and cultured for 48 h at 37°C in a 5% CO_2_ atmosphere. After incubation with CCK-8 solution (10 ml/well) for 1 h, the absorbance value at 450 nm was measured using a micro plate reader and analyzed at 24 h intervals [24], while the 650 nm served as the reference wavelength. All experiments were performed in triplicate, and the results were representative of three individual experiments.

### Western Blotting Analysis

Western blotting was performed to determine Wip1 expression in human ccRCC tissues and cell lines. Briefly, total protein was extracted using RIPA Buffer (Sigma, St Louis, MO, USA), and protein concentrations were quantified by BCA Protein Assay Kit (Boster Ltd., Wuhan, China). Equal amounts of harvested protein samples were resolved on a 10% SDS–PAGE and transferred to PVDF membranes (Millipore Corporation, Billerica, MA, USA). The blotted membranes were blocked with 5% BSA in TBST for 2 h at room temperature, and incubated with the indicated primary antibodies overnight at 4°C. The rabbit polyclonal anti-Wip1 (Santa Cruz Biotechnology, Santa Cruz, CA) was used at the dilution of 1∶400, whileβ-actin (Cell Signaling Technology, Beverly, MA) was used as a loading control. The membranes were washed and then incubated with appropriate horseradish peroxidase (HRP)-conjugated secondary antibodies (Amersham, GE Healthcare, Little Chalfont, UK) for another 2 h at room temperature. Immunoblots were visualized by enhanced chemiluminescence using Supper Signal West Pico Trail Kit (Pierce Biotechnology, Rockford, IL, USA) according to the manufacturer's instructions.

### RNA Extraction and Quantitative Real-Time PCR Assays

Total RNA was extracted and purified from frozen tissue samples and cell lines using the Trizol reagent (Invitrogen Corporation, Carlsbad, CA, USA) according to the manufacturer's instructions. cDNA was prepared as previously described and used as a template for quantitative real-time polymerase chain reaction (PCR) performed on an ABI StepOnePlus Real-Time PCR System using the SYBR Green Real-time PCR Master Mix (Applied Biosystems Inc., Foster City, CA, USA) [18]. The sequences of gene specific primers for Wip1 (forward, 5′- GAAGGATGACTTTGTCAG -3′; reverse, 5′- CCCAGACTTGTTCATTAC-3′) andβ-actin (forward, 5′- CATCCTGCGTCTGGACCTGG -3′; reverse, 5′- TAATGTCACGCACGATTTCC -3′) were designed using NCBI Primer-BLAST. The cycle threshold (Ct) values were standardized to Ct values of β-actin, and fold difference in occupancy was calculated as follows: Fold difference  = 2^−ΔΔCt^.

### Statistical Analyses

Statistical analyses were performed using SPSS software version 16.0 for Microsoft Windows (SPSS Inc., Chicago, IL, USA). Group differences for qualitative variables were analyzed using chi-square test, and tumor-free survival of patients was stratified using the Kaplan-Meier method and statistically analyzed using the log rank statistic. Univariate and multivariate analyses using Cox proportional hazard models were conducted to measure correlations between clinicopathological factors and tumor-free survival. A p<0.05 was considered statistically significant.

## Results

### Expression of Wip1 Increased in Human Primary Ccrcc Tissues and RCC Cell Lines

**Figure 1:**
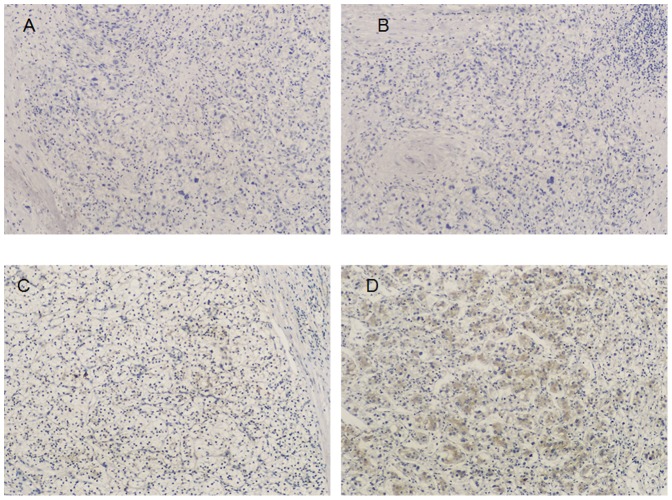
Expressions of Wip1 in human ccRCC tissues and matched non-cancer tissues. (A, B) Negative or low level staining of Wip1 in paired normal tissue and in small part of ccRCC tissues; (C) Moderate staining of Wip1 in tumor tissue; (D)Strong staining in majority of ccRCC tissue; Magnification: 400×.

**Figure 2:**
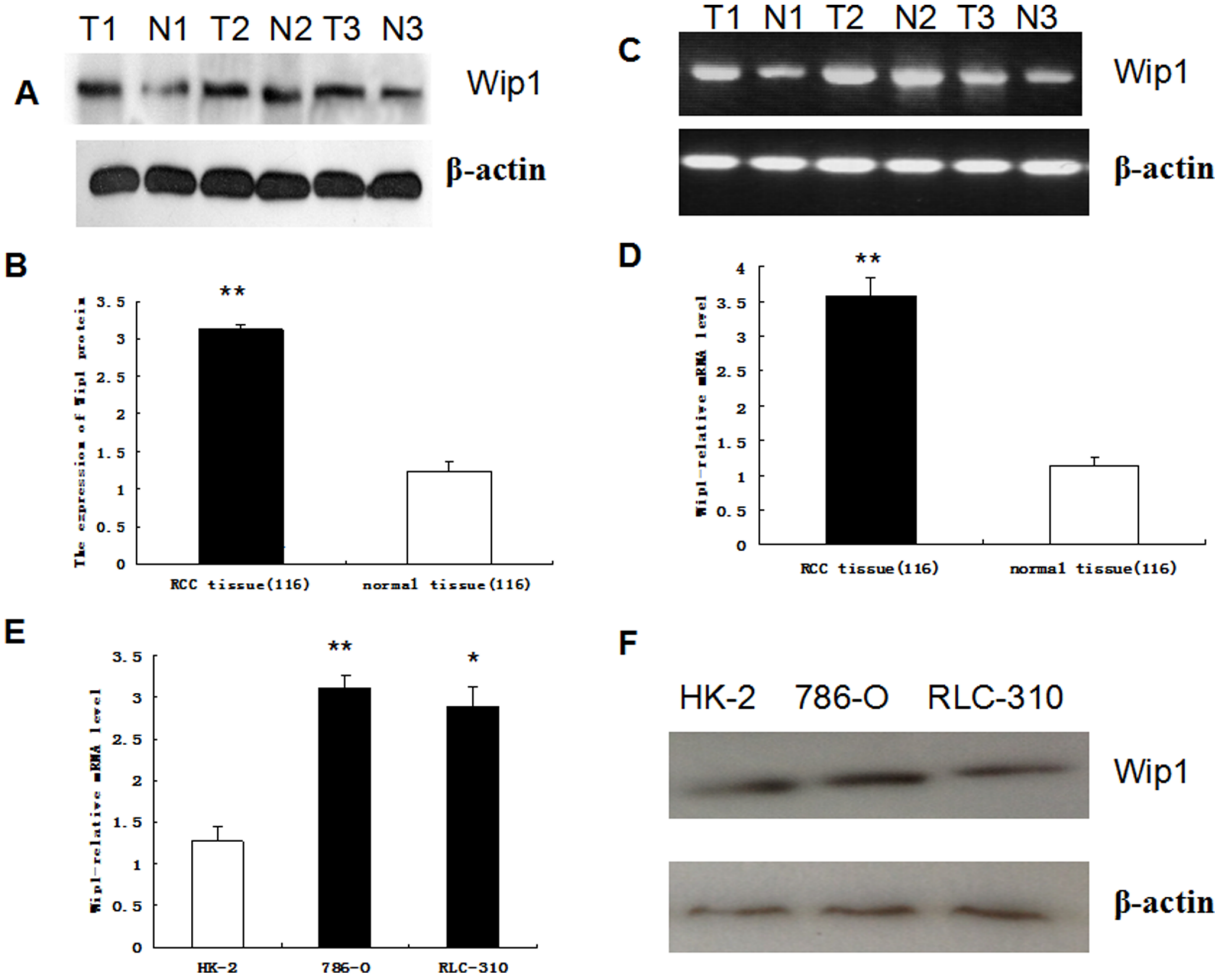
High expression of Wip1 in RCC Tissue and cell lines. Expressions of Wip1 protein and mRNA in representative pairs of matched normal renal tissues (N) and ccRCC tissues (T) were assessed by western blotting (A, B) and qRT-PCR(C, D), respectively. Wip1 expression in immortalized normal human proximal tubule epithelial cell line HK-2 and two types of RCC cell lines (786-O, RLC-30) was determined by qRT-PCR (E),western blotting (F) respectively. β-actin served as a loading control. Data were shown as mean±SD, **P<0.001, *P<0.05.

To investigate the expression and distribution of Wip1 protein and mRNA in ccRCC tissues, the immunohistochemistry, qRT-PCR and western blotting analysis were initially performed in 116 pairs of human ccRCC and matched normal tissues, respectively. Wip1 staining was shown mainly in the cytoplasm of cancer cells and a combination of the nucleus and cytoplasm, while positive signals were very rare in normal renal tissues (Fig. 1A) and in small part ccRCC tissues (Fig. 1B). A significant upregulation of Wip1 protein was observed in the ccRCC tissues (Fig. 1C, D). To further confirm these observations, we investigated the expression of Wip1 in ccRCC tissues and paired tissues using western blotting. Our data clearly indicated that the cancer tissue had a drastic increase of Wip1 expression as compared with the corresponding normal tissues (Fig. 2A, B). In line with this, the expression of Wip1 mRNA was found significantly higher in ccRCC specimens than that in normal renal tissues (P<0.001; Fig. 2C, D). In addition, the levels of Wip1 mRNA and protein were determined in three types of cell lines, including 786-O, RLC-310 and HK-2. The expressions of both Wip1 mRNA and protein were significantly upregulated in RCC cell lines as compared with that in the immortalized normal human proximal tubule epithelial cell line HK-2 (Fig. 2E–F), which indicated Wip1 expression was associated with the aggressive phenotypes of RCC cells.

### Wip1 Knockdown Inhibited the Invasive Ability of RCC Cells *In Vitro*

**Figure 3:**
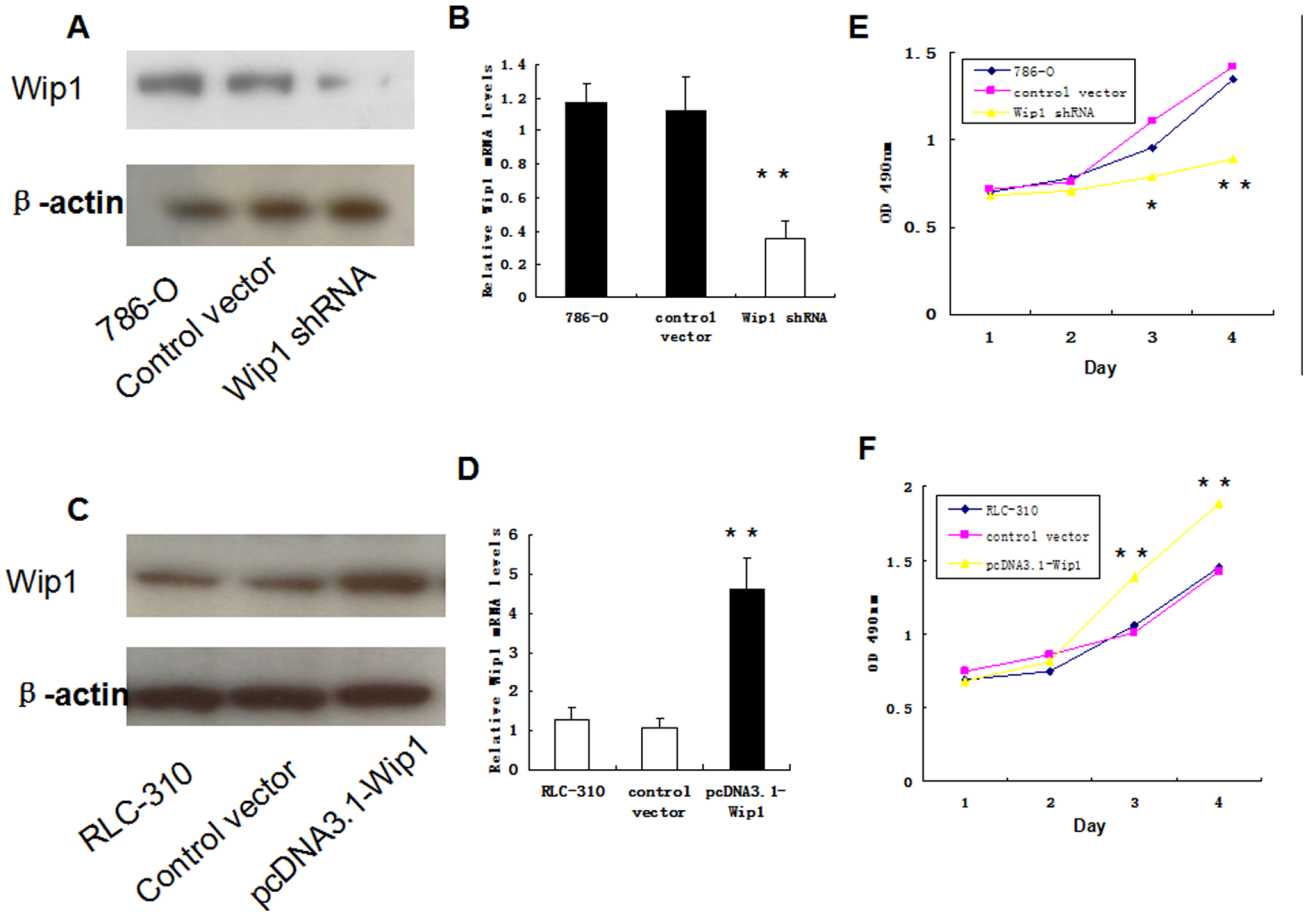
Effects of suppression and overexpression of Wip1 on RCC cell line 786-O and RLC310. The 786-O cells were transfected with control-shRNA or Wip1-shRNA, while RLC-310 cells were treated with pcDNA3.1 control vector or pcDNA3.1-Wip1, and nontransfected 786-O and RLC-310 cells were used as blank controls respectively. As compared to control groups, both mRNA and protein levels of Wip1were significantly decreased in 786-O cells after Wip1 knockdown (A, B), and remarkably increased in RLC-310 cells with Wip1 overexpression (C, D). Evaluation of cell proliferation by CCK-8 assays showed that down-regulation of Wip1 dramatically inhibited the cell proliferation rate of 786-O cells (E), whereas overexpression of Wip1 significantly promoted the growth of RLC-310 cells (F). Data were shown as mean ± SD (n = 3). **P<0.001, *P<0.05.

As shown in Fig. 2E, the 786-O, RLC-310 cells had the highest level of Wip1 expression among the three types of cell lines, Therefore, we next assayed the alterations to the invasive ability in 786-O and RLC-310 cells using Matrigel invasion assays. First we were selected for Wip1 gene silencing to study the effect of Wip1 knockdown on invasive phenotype and growth of renal cancer cells in vitro. After Wip1-specific shRNAs was transfected into 786-O cells, the expression of Wip1 protein was significantly inhibited (Fig. 3A). Moreover, the level of Wip1 mRNA was also remarkably reduced as compared with those transfected with control shRNA (P<0.05; Fig. 3B). These results indicated that Wip1-specific shRNAs could effectively and specifically knockdownWip1 expression at both transcriptional and translational levels.

**Figure 4:**
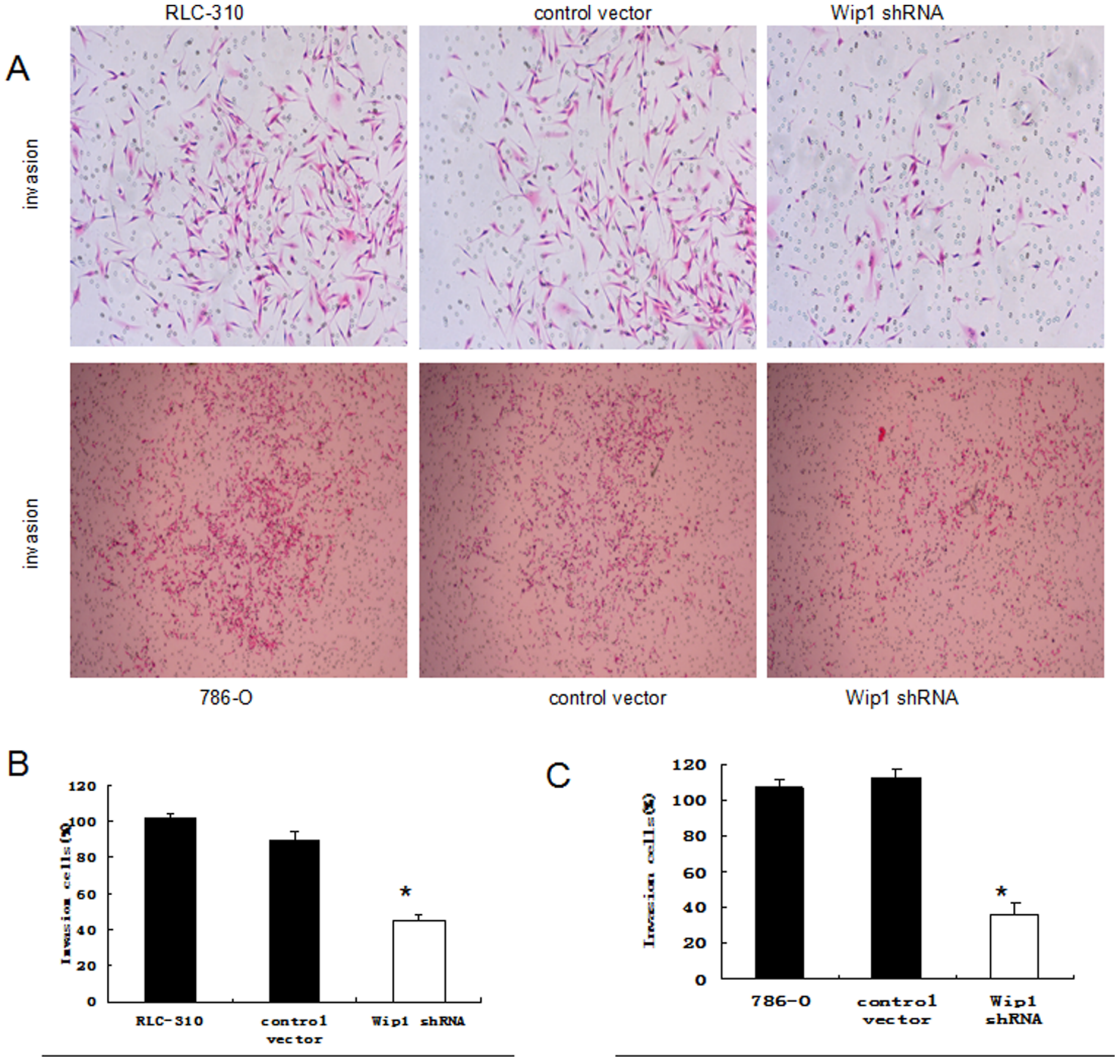
Wip1 knockdown inhibited the invasive ability of RCC cells in vitro. A, Representative images of the invaded 786-O and RLC-30 cells in Matrigel invasion assays were taken at ×200 magnification. B, C: Numbers of the invaded 786-O and RLC-30 cells per microscopic field. Data are presented as the means ± SD from three independent experiments. * p<0.05.

To further determine the effect of Wip1 on the migration, invasion and proliferation of renal cancer cells in vitro, the transwell migration and invasion assays and CCK-8 assay were carried out with renal cancer cells cells, respectively. As shown in Fig. 4A, B, the numbers of parental and control RLC-310 cells that passed through the Matrigel-coated membrane were significantly higher ((97±27 and 102±37, respectively) than those of RLC-310 cells that underwent Wip1 shRNA silencing (45±26) (p<0.05). Similar results were also obtained in 786-O cells (101±17, 117±29 versus 40±27 (p<0.05); Fig. 4A, C). These results vividly demonstrated that WIP1 mediated the invasiveness of renal cancer cells in vitro.

### Overexpression of Wip1 Augments the Migration Ability of RCC Cells *In Vitro*

**Figure 5:**
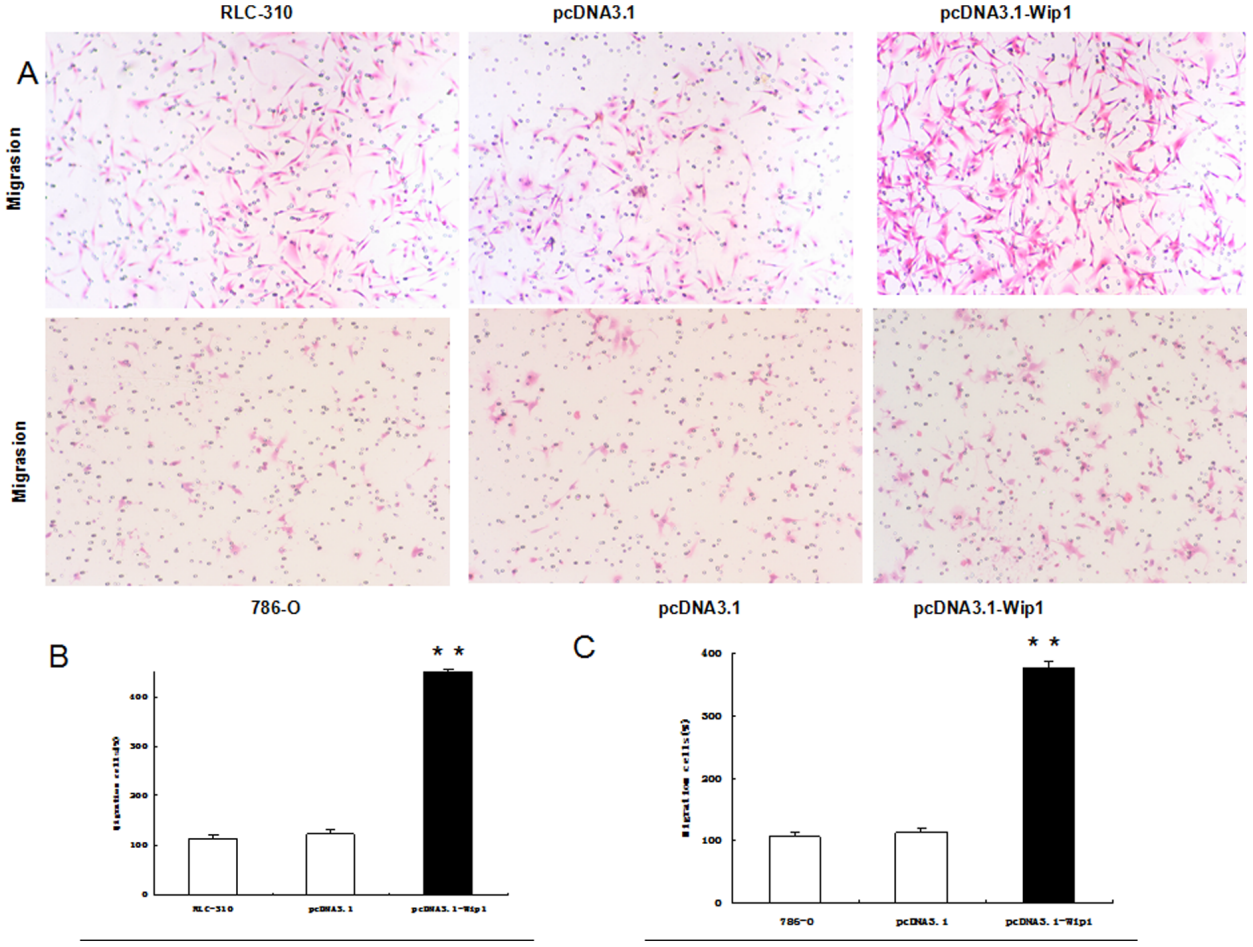
Overexpression of Wip1 augments the migration ability of RCC cells in vitro. A, Representative images of the migrate 786-O and RLC-30 cells in Matrigel invasion assays were taken at ×200 magnification. B, C: Numbers of the invaded 786-O and RLC-30 cells per microscopic field. Data are presented as the means ± SD from three independent experiments. * * p<0.001.

We next examined whether ectopic expression of Wip1 was sufficient to promote the migration capability of RCC cells. After pcDNA3.1-Wip1 was stably transfected into the 786-O, RLC-310 cells, both the mRNA and protein levels of Wip1 were dramatically up-regulated as compared with those in the nontransfected group (P<0.001; Fig. 3C–D), Furthermore, the migration capability was significantly increased to approximately 4.5- and 4.0-fold in vitro (P<0.001; Fig. 5A-C), respectively. However, no significant difference was observed between the pcDNA3.1 empty vector-transfected group and the control group. These data confirmed that ectopic expression of Wip1 in 786-O, RLC-310 cells by pcDNA3.1-Wip1 vector could promote their capability of migration. Additionally, we employed the CCK-8 cell proliferation assay to test effects of Wip1 overexpression on cell growth of 786-O and RLC-310. As compared with control cells and cells transfected with pcDNA3.1 empty vector, the pcDNA3.1-Wip1-transfected cells showed a greater proliferation ratio and higher relative absorbance value (*P<0.05, **P<0.001; Fig. 3E, F). Taken together, these results suggested that overexpression of Wip1 could facilitate the growth and aggressive phenotype of renal cancer cells in vitro.

### Immunohistochemical Analysis of Wip1 Expression in Ccrcc Clinical Samples and Its Relationships to Clinicopathological Features

The relationships between Wip1 expression and clinicopathological parameters of ccRCC were summarized in Table 1. A total of 77 (66.4%) cases showed high levels of Wip1 expression (score 3–6). The increased expression of Wip1 protein showed a significant correlation with depth of invasion (P<0.001) and lymph node status (P<0.001). Furthermore, our data also demonstrated that high Wip1 expression was dramatically associated with Fuhrman grade (P<0.001) and distant metastasis (P = 0.001), which served as an important prognostic marker for patients with ccRCC. However, no significant association was found between Wip1 expression and other clinicopathologic features, including age, gender, primary tumor size, and TNM stage.

### Association of Wip1 Expression with Survival of Ccrcc Patients

**Figure 6:**
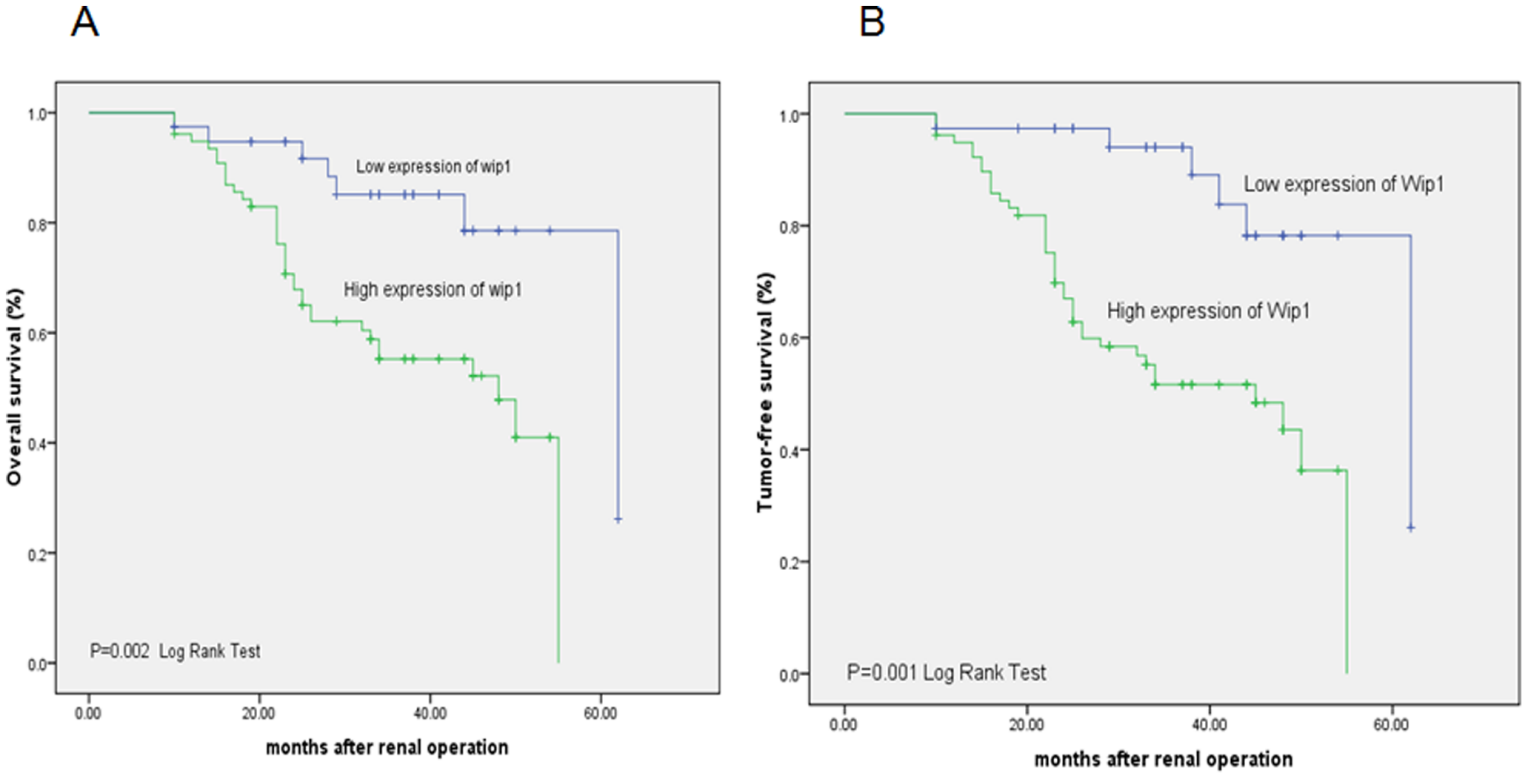
Kaplan–Meier survival curves for Wip1 expression. A, overall survival B, tumor-free survival. The patients with positive Wip1 expression displayed a significant worse outcome compared to the patients with negative Wip1 expression. (P = 0.002, log-rank test) and (P = 0.001, log-rank test).

Survival analysis was performed in all the patients and follow-up data were collected. All patient follow-ups ended in November 30, 2011 after a revisit time of 62 months. Among all cases, 72 were still alive at this time and 44 were dead. A survival curve was drawn. Patients were divided into two groups according to Wip1 expression level. There were 77 individuals with high levels of Wip1 expression, among whom 41 were still alive and 36 were dead. The survival rate was 53.2%. There were 39 individuals with low levels of Wip1 expression, among whom 31 were still alive and 8 were dead. The survival rate was 79.5%. Patients with low levels of Wip1 expression had significantly higher 5-year survival rates than those with high levels of Wip1 expression group (P = 0.002) (Figure 6A). During the 62 months of follow-up, 70 cases were non-recurrent and 46 cases were recurrent. A survival curve was drawn. Among the 77 individuals with high levels of Wip1 expression, 38 cases were non-recurrent and 39 were recurrent, producing a non-recurrence ratio of 49.4%. Among the 39 individuals with low levels of Wip1 expression, 32 cases were non-recurrent and 7 were recurrent, producing a non-recurrence ratio of 82.1% (P = 0.001) (Figure 6B). The uni- and multivariate analyses indicated that expression of Wip1 was independent predictors for tumor-free survival of ccRCC patients (Table 2). The univariate analysis showed that depth of invasion, grade, lymph node/distant metastasis were associated with poor survival of ccRCC patients, while the multivariate analysis showed that tumor grade, depth of invasion, distant metastasis and Wip1 expression were independent predicators for survival of ccRCC patients (Table 2).

**Table 2:** Univariate and multivariate analyses of clinicopathological factors affecting overall survival of ccRCC patients.

Characteristics	Univarate analysis		Multivariate analysis	
	HR(95% CI)	P-value	HR(95% CI)	P-value
Age(yers)				
<50	1			
≥50	1.03(0.66–1.87)	0.412		
gender				
Male	1			
Female	0.97(0.63–1.76)	0.349		
Tumor size (cm)				
≤4.0	1			
4.1∼7.0	1.13(0.85–2.01)	0.512		
>7.1	0.82(0.53–1.48)	0.217		
Depth of invasion				
T1+T2	1			
T3+T4	1.76(0.89–3.31)	0.001	1.45(0.72–2.79)	**0.032**
Symptoms of diagnosis				
Incidental	1			
Symptoms	1.92(1.66–3.35)	0.074		
Fuhrman grade				
G1–2	1			
G3–4	2.92(1.56–5.73)	0.001	2.45(1.25–3.95)	**0.011**
TNM stage				
I+II	1			
III+IV	2.34(1.53–3.45)	0.063		
Lymph node status				
Negative	1			
Positive	1.82(1.24–2.97)	0.008	1.62(0,97–2.16)	0.075
Distant metastasis				
Absent	1			
Present	2.26(1.15–4.86)	0.002	1.96(1.20–3.58)	**0.023**
Wip1 expression				
Low	1			
High	3.12(1.46–5.23)	0.003	2.47(1.72–4.67)	**0.027**

## Discussion

Currently an effective treatment modality for ccRCC is surgical lesion resection, accompanied by chemotherapy and/or radiotherapy before and after surgery. However, the survival rate with this strategy is not very satisfactory [5]. Therefore, more attention should be directed at the importance of early detection of kidney cancer, and the choice of treatment strategies should be carefully weighed for individual. Wild-type p53-induced phosphatase 1 (Wip1 or PPM1D) is involved in DNA repair and cell cycle checkpoint pathways and Wip1 is frequently amplified or overexpressed in different human cancers, promoting tumor growth by switching off major checkpoint kinases and p53 [10], [17], [18], [19], [20]. Thus, this study investigated Wip1 expression in RCC tissue specimens and associated it with clinicopathological data and survival of patients in order to assess Wip1 as a biomarker for RCC.

In the current study, we provided evidence for the emerging links that Wip1 was overexpressed mainly with a cytoplasm pattern in human ccRCC specimens, which was significantly associated with aggressive phenotypes of tumor cells. In keeping with these findings, our data further revealed that depletion of Wip1 suppressed the proliferation and aggressiveness of renal cancer cells with higher invasive activities in vitro; on the contrary, ectopic introduction of Wip1 resulted in increased growth and aggressive phenotype of renal cancer cells. In addition, the survival analysis in the current study showed that the tumor-free/overall survival rate for patients in the Wip1-positive expression group was significantly lower than that of the Wip1-negative group. The Cox multivariate analysis indicated that positive Wip1 expression; symptoms of diagnosis, Fuhrman grade and distant metastasis were significant prognostic predictors. These results suggest that Wip1 is crucially implicated in the carcinogenesis and invasion of RCC.

Previous studies indicate that when DNA damage occurs, ATM causes Chk2 phosphorylation to prevent tumorigenesis. Wip1 combines with Chk2 to dephosphorylate and inactivate Chk2, leading to tumorigenesis. In vivo and in vitro experiments confirmed that Wip1 could also contribute to tumorigenesis by inhibiting Chk1 dephosphorylation and inactivation [8]. Further studies demonstrated that Wip1 high expression disrupted the homeostasis maintained by the p38MA PK-p53-Wip1 pathway, caused downstream Wnt-p53 inactivation through p38MAPK dephosphorylation, and promoted the development of malignant breast cancer in humans by reducing p16 protein levels [21]. In recent years, increasing bodies of researches have shown that Wip1 is highly expressed in neuroblastoma, pancreatic cancer, lung cancer, bladder cancer, liver cancer, ovarian cancer, and breast cancer [10], [11], [12] [19], [20], [22]. Wip1 alters the balance between pro-apoptotic and anti-apoptotic proteins and switches off DNA damage checkpoint responses by dephosphorylating certain proteins involved in DNA repair and cell cycle checkpoints [11], [18],and overexpression of Wip1 is an indicator for some patients at high risk of tumor-related death [23]. Buss et al.. [24] recently showed that WIP1 may play a role in invasion and possibly metastasis of medulloblastoma, and thus Wip1 protein had been proposed as a novel biological marker for medulloblastoma progression and metastasis. These results correspond well with our present findings, which provided additional evidence for the concept that Wip1 plays a crucial role in promoting tumor growth, invasion and metastasis of various types of malignancies, and may also have a potential value of being a molecular target for cancer therapy.

In order to provide further support that Wip1 contributes to the development and progression of renal cancer, several cell lines (786-O, RLC-310 and HK-2) were employed for gain and loss of function experiments. In light of our results, the levels of Wip1 in 786-O cells and RLC-310 cells were high than HK-2. Based on these findings, we effectively down-regulated Wip1 expression in 786-O cells by Wip1-shRNA in vitro, and our data indicated that proliferation and invasiveness of stable transfected cells were significantly decreased. On the contrary, overexpression of Wip1 in RLC-310 cells mediated by pcDNA3.1-Wip1 resulted in the increased growth and aggressive phenotype in vitro. Taken together, both gain and loss of function experiments further confirmed that upregulation of Wip1 could facilitate the proliferation and aggressiveness of renal cancer cell lines in vitro, which was consistent with our data from the immunohistochemical analysis using the clinical ccRCC samples.

Inhibiting positive regulators of cell proliferation, activating tumor suppressor pathways and inducing apoptosis are primary intervention strategies in modern cancer therapy. Among positive regulators of proliferation, Wip1 complements different oncogenes in the transformation of wild-type MEFs [17]. Recent reports have indicated that Wip1 overexpression in transgenic mice promotes cell transformation and accelerates cancer progression [25]. In contrast, Wip1-knockout mice are resistant to mammary cancer. In addition, even when tumors form in these mice, the tumor cells exhibit a low proliferation potential [26]. Our results show overexpression of Wip1 augments the growth and aggressive phenotype of renal cancer cells in vitro. On the other hand, we showed that Wip1 was highly expressed in the majority of malignant ccRCC and that Wip1 protein levels correlated with increasing tumor grades, depth of invasion, lymph node status and distant metastasis. The Uni-multivariate analysis indicated that positive Wip1 expression, depth of invasion, fuhrman grade and distant metastasis were significant prognostic predictors. Patients with positive levels of Wip1 expression had significantly lower 5-year overall/tumor-free survival rates than those with low levels of Wip1 expression group. The results suggest that Wip1 plays an important role in tumorigenesis and progression of RCC.

In summary, our data provided a basis for the concept that Wip1 expression was significantly upregulated in ccRCC tissues and RCC cell lines, which might be associated with adverse biologic behavior of cancer cells to promote the tumorigenesis and progression of RCC. More importantly, significant correlations between Wip1 expression and survival of ccRCC patients were observed in the present study. This may be potentially significant to suggest that targeting of the Wip1 pathway may constitute a novel treatment modality for the prevention of ccRCC progression. However, the mechanism responsible for Wip1 in tumorigenesis and the biological functions merits further evaluation.
