# Correction: Overexpression of Wip1 Is Associated with Biologic Behavior in Human Clear Cell Renal Cell Carcinoma

**DOI:** 10.1371/journal.pone.0122427

**Published:** 2015-03-16

**Authors:** The PLOS ONE Staff

The third author’s name is spelled incorrectly. The correct name is: Weiqing Han.
